# CrossTalk opposing view: CNNM proteins are not Na^+^/Mg^2+^ exchangers but Mg^2+^ transport regulators playing a central role in transepithelial Mg^2+^ (re)absorption

**DOI:** 10.1113/JP275249

**Published:** 2018-01-31

**Authors:** Francisco J. Arjona, Jeroen H. F. de Baaij

**Affiliations:** ^1^ Department of Physiology Radboud Institute for Molecular Life Sciences, Radboud University Medical Center Nijmegen The Netherlands

**Keywords:** CNNM2, CNNM4, magnesium, ion transport, exchanger, transporter

## Introduction

Magnesium (Mg^2+^) is indispensable for many physiological processes in the cell impacting organ function (de Baaij *et al*. 2015). Intracellular concentrations of free Mg^2+^ are maintained within a narrow range (0.5–1.2 mm) through tightly regulated Mg^2+^ influx and efflux mechanisms (Ebel & Gunther, 1980). Inhibition of Mg^2+^ efflux by substitution of extracellular Na^+^ by choline in mammalian cells indicates that Mg^2+^ extrusion is Na^+^ dependent (Guther *et al*. 1984). Thus, it is postulated that Mg^2+^ efflux occurs via a Na^+^/Mg^2+^ exchanger, this notion being corroborated in a large number of studies in different cell models (Romani, 2007).

Under the premise that differential gene expression is involved in the maintenance of cellular Mg^2+^ homeostasis, microarray analyses in epithelial cells exposed to low extracellular Mg^2+^ concentrations have been classically used to designate proteins involved in Mg^2+^ transport (Quamme, 2010). One of the protein families identified with this approach is the CNNM family. CNNM proteins have been proposed to facilitate epithelial Mg^2+^ extrusion since at least two members of this family, CNNM2 and CNNM4, localize in the basolateral membrane of epithelial cells where apical‐to‐basolateral Mg^2+^ transport occurs (Stuiver *et al*. 2011; Yamazaki *et al*. 2013). However, in this review of the literature, we will demonstrate that the evidence supporting this hypothesis is controversial and several findings suggest that CNNMs cannot function as Na^+^/Mg^2+^ exchangers.

## Topology

**Figure 1:**
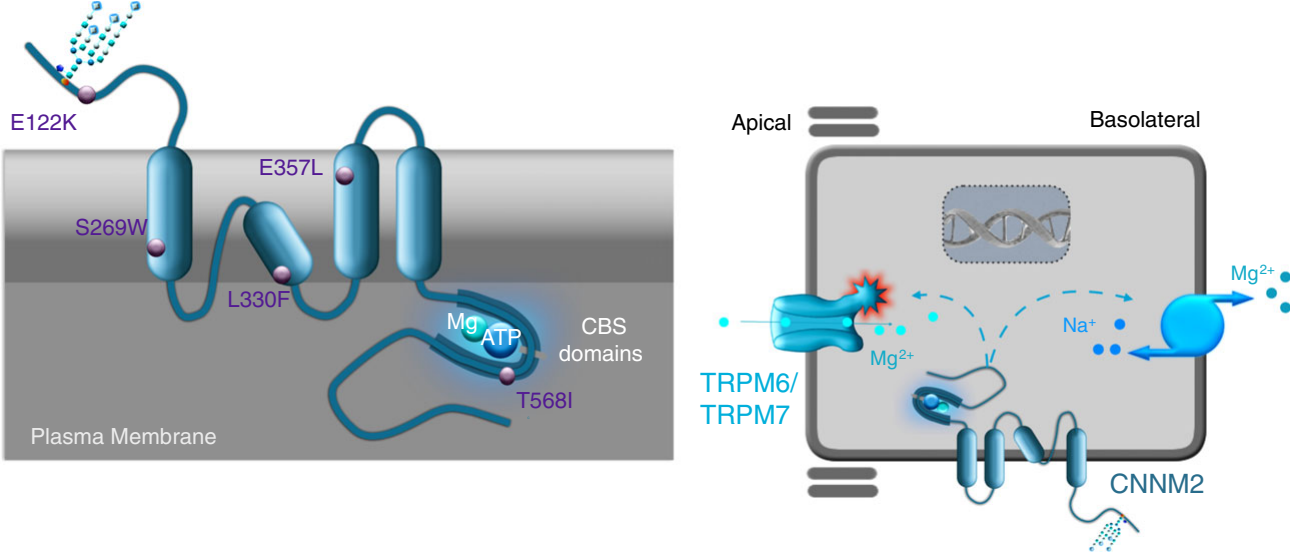
Membrane topology and subcellular localization of CNNM2 The left panel indicates the membrane topology of CNNM2 showing three full membrane‐spanning domains and a large intracellular C‐terminus containing two CBS modules that bind Mg‐ATP. Reported mutations have been indicated by purple dots. The right panel shows the basolateral localization of CNNM2 in the distal convoluted tubule kidney cell.

The CNNM family (CNNM1–4) shares a high homology with the bacterial CorC protein, which is related to the maintenance of Mg^2+^ and Co^2+^ homeostasis (Hmiel *et al*. 1989). However, CorC requires CorA to function as a cation transporter and does not transport Mg^2+^ itself (Gibson *et al*. 1991). Within the CNNM family, membrane topology studies have demonstrated that CNNM2 is composed of dimers of three transmembrane domains (Fig. 1) (de Baaij *et al*. 2012). This number of transmembrane domains is significantly lower than that in typical Mg^2+^ channels/transporters such as TRPM6/M7 (tetramers of 6 transmembrane domains), or SLC41A1/A3 (dimers of 11 transmembrane domains) (Voets *et al*. 2004; Bates‐Withers *et al*. 2011; Kolisek *et al*. 2012; de Baaij *et al*. 2016). Additionally, a putative pore facilitating cation transport is unlikely to be present in CNNM2 since the re‐entrant loop in its tertiary structure does not completely span the cell membrane and therefore does not facilitate the formation of a typical pore (Fig. 1) (de Baaij *et al*. 2012).

A common feature of CNNM proteins is the presence of intracellular cystathionine β‐synthase (CBS) domains (de Baaij *et al*. 2012). The CBS domains are the main site of regulation, as has recently been demonstrated by a number studies showing the interaction with phosphatases of the regenerating liver (PRL) proteins (Hardy *et al*. 2015; Gulerez *et al*. 2016; Kostantin *et al*. 2016). CBS domains regulate protein function upon binding Mg‐ATP (Corral‐Rodriguez *et al*. 2014; Hirata *et al*. 2014). Most intracellular Mg^2+^ is bound to ATP. Intracellular Mg‐ATP changes in concentration can be sensed by CNNM proteins and may regulate their function(s). Although the exact role between ATP production and Mg^2+^ concentrations is unknown, an interesting hypothesis is that CNNM proteins function as indirect sensors of intracellular Mg^2+^ concentrations.

## Function

Since its identification in 2003, the function of CNNM proteins has been subject to debate (Wang *et al*. 2003). Because of their homology to cyclin proteins, CNNMs were initially hypothesized to have a role in cell cycle progression. However, a cyclin function was never demonstrated. For years, CNNM2 has been the main focus of functional CNNM experiments, as the protein was identified in renal distal convoluted tubule (DCT) cells (Goytain & Quamme, 2005; Stuiver *et al*. 2011; de Baaij *et al*. 2012). In the DCT, Mg^2+^ is actively reabsorbed and urinary Mg^2+^ excretion is determined. In human studies, TRPM6 has been established as the Mg^2+^ uptake mechanism in this nephron segment (Schlingmann *et al*. 2002). However, recent studies in mice show that TRPM7 may be equally important in determining renal Mg^2+^ excretion (Chubanov *et al*. 2016). The protein mediating Mg^2+^ extrusion still has to be identified.

As a result, several groups have examined the potential Mg^2+^ transport properties of CNNM2 using electrophysiology in a multitude of cell models. In 2005, Quamme and his team showed CNNM2‐mediated Mg^2+^ currents in *Xenopus laevis* oocytes with a *K*_m_ for Mg^2+^ of 0.56 ± 0.05, establishing CNNM2 as a Mg^2+^ transporter (Goytain & Quamme, 2005). In the DCT, luminal Mg^2+^ concentrations range between 0.2 and 0.7 mm Mg^2+^ (Dai *et al*. 2001), supporting CNNM2‐mediated Mg^2+^ uptake. However, in mammalian cells, these findings could not be reproduced by two independent research groups (Stuiver *et al*. 2011; Sponder *et al*. 2016), although a minor Mg^2+^‐dependent Na^+^ current was detected in HEK293 cells overexpressing CNNM2 (Stuiver *et al*. 2011; Yamazaki *et al*. 2013). From 2013 onwards, CNNM4 function has also been examined, as it was shown to be expressed in the colon and to be involved in cancer progression (Yamazaki *et al*. 2013; Funato *et al*. 2014). In electrophysiological examinations of CNNM4‐expressing cells, no Mg^2+^ currents were detected (Yamazaki *et al*. 2013). Because electrophysiology does not permit the detection of electroneutral Mg^2+^ transport, this led several authors to suggest that CNNM proteins might be Na^+^‐dependent Mg^2+^ exchangers at a 2:1 (Na^+^:Mg^2+^) ratio.

To tackle this hypothesis, alternative experiments were staged. Several groups have aimed to measure CNNM activity using fluorescent Mg^2+^ probes, resulting in a large pile of conflicting data. First, using the Mg^2+^‐sensitive fluorescent Magnesium Green indicator, the group of Miki showed that overexpression of CNNM4 or CNNM2 decreased cellular Mg^2+^ concentrations (Yamazaki *et al*. 2013; Funato *et al*. 2014). Their data suggest that the Mg^2+^ efflux is Na^+^ dependent, supporting the exchange theory. Second, Tremblay and colleagues demonstrated CNNM3‐dependent Mg^2+^ uptake using Mag‐Fura‐2 (Hardy *et al*. 2015; Kostantin *et al*. 2016). As their experiments are performed in a physiological 140 mm Na^+^‐containing buffer, this contradicts Na^+^ dependency. Third, Kolisek's group could not detect CNNM2‐mediated Mg^2+^ influx nor efflux, questioning the ability of CNNMs to transport Mg^2+^ (Sponder *et al*. 2016). Several important limitations of these experiments have to be considered. All experiments were performed in non‐physiological Mg^2+^ concentrations, including loading steps using 10 mm up to 40 mm Mg^2+^. Moreover, the Mg^2+^ probes are notoriously difficult to work with as they have dissociation constants above physiological Mg^2+^ concentrations, as well as sensitivity to Ca^2+^ (Szmacinski & Lakowicz, 1996). Additionally, Magnesium Green is a non‐ratiometric probe, which means that the experiments are extremely sensitive to osmolality changes.

To overcome these hurdles, we have recently developed a stable isotope assay using ^25^Mg^2+^ and mass spectrometry. This set‐up measures cellular Mg^2+^ uptake using the physiological condition of 1 mm Mg^2+^. Our ^25^Mg^2+^ uptake experiments, performed in HEK293 cells overexpressing CNNM2, demonstrate that CNNM2 increases cellular Mg^2+^ uptake rather than Mg^2+^ efflux (Arjona *et al*. 2014). This conclusion is illustrated by the fact that inhibition of Mg^2+^ efflux through Quinidin, an inhibitor of Na^+^/Mg^2+^ exchange, did not affect CNNM2‐mediated ^25^Mg^2+^ cellular accumulation. Interestingly, this uptake is independent of Na^+^, as replacing Na^+^ with NMDG had no effect, nor did ouabain, an inhibitor of the Na^+^/K^+^‐ATPase (Arjona *et al*. 2014). However, the CNNM2‐dependent Mg^2+^ uptake was completely abolished by using 2‐aminoethoxydiphenyl borate (2‐APB), which is a known inhibitor of TRPM7 (Chokshi *et al*. 2012; Arjona *et al*. 2014).

Altogether, the functional experiments in a wide range of models and techniques show that CNNM2 is not a Na^+^/Mg^2+^‐exchanger, as the Mg^2+^ transport is independent of Na^+^ and can be bi‐directional (influx and efflux). Moreover, the discrepancy between the different models shows that CNNM2 function is largely dependent on other proteins present in the cell. This is suggestive of a regulating function rather than a transport function. Given that the CNNM2‐dependent ^25^Mg^2+^ uptake could be blocked by 2‐APB, TRPM7, a channel involved in Mg^2+^ homeostasis, is an interesting candidate for regulation by CNNM2 (Paravicini *et al*. 2012).

In an *in vivo* context, dysfunctional CNNM2 has been linked to defects in renal Mg^2+^ reabsorption in mice and zebrafish (Arjona *et al*. 2014; Funato *et al*. 2017). In addition, CNNM4 knockout in mice results in impaired intestinal Mg^2+^ absorption (Yamazaki *et al*. 2013). Thus, though CNNM proteins are not Mg^2+^ transporters, they regulate the body Mg^2+^ balance.

## Conclusion and perspective

Hence, the following evidence supports that CNNM proteins are not Na^+^/Mg^2+^ exchangers: (i) CNNM proteins do not comply with the consensual topological attributes of a typical Mg^2+^ transporter, i.e. abundant transmembrane domains and a cation pore formed by membrane‐spanning domains; (ii) conflicting functional data of CNNM‐mediated Mg^2+^ extrusion between different cellular models indicate that the Mg^2+^ extrusion function of CNNM proteins depends on the Mg^2+^ channels/transporters specifically expressed by each cellular model; and (iii) CNNM2‐mediated Mg^2+^ extrusion in epithelial cells is not dependent on Na^+^ in a series of experiments. Altogether, this shows that CNNM proteins are not Na^+^/Mg^2+^ exchangers. Future research should focus on the identification of the protein partners of CNNMs to elucidate the CNNM‐dependent regulation of Mg^2+^ transport.

## Call for comments

Readers are invited to give their views on this and the accompanying CrossTalk articles in this issue by submitting a brief (250 word) comment. Comments may be submitted up to 6 weeks after publication of the article, at which point the discussion will close and the CrossTalk authors will be invited to submit a ‘LastWord’. Please email your comment, including a title and a declaration of interest, to jphysiol@physoc.org. Comments will be moderated and accepted comments will be published online only as ‘supporting information’ to the original debate articles once discussion has closed.

## Additional information

### Competing interests

None declared.

### Author contributions

Both authors have approved the final version of the manuscript and agree to be accountable for all aspects of the work. All persons designated as authors qualify for authorship, and all those who qualify for authorship are listed.

### Funding

This work was financially supported by grants from the Netherlands Organization for Scientific Research (NWO VENI 016.186.012) and the Dutch Kidney Foundation (Kolff 14OKG17).
